# Characterisation of a niche-specific excretory–secretory peroxiredoxin from the parasitic nematode *Teladorsagia circumcincta*

**DOI:** 10.1186/s13071-019-3593-6

**Published:** 2019-07-10

**Authors:** Daniel R. G. Price, Alasdair J. Nisbet, David Frew, Yvonne Bartley, E. Margaret Oliver, Kevin McLean, Neil F. Inglis, Eleanor Watson, Yolanda Corripio-Miyar, Tom N. McNeilly

**Affiliations:** Moredun Research Institute, Pentlands Science Park, Edinburgh, EH26 0PZ UK

**Keywords:** Peroxiredoxin, Excretory–secretory, Niche, Anti-oxidant, Peroxidase

## Abstract

**Background:**

The primary cause of parasitic gastroenteritis in small ruminants in temperate regions is the brown stomach worm, *Teladorsagia circumcincta*. Host immunity to this parasite is slow to develop, consistent with the ability of *T. circumcincta* to suppress the host immune response. Previous studies have shown that infective fourth-stage *T. circumcincta* larvae produce excretory–secretory products that are able to modulate the host immune response. The objective of this study was to identify immune modulatory excretory–secretory proteins from populations of fourth-stage *T. circumcincta* larvae present in two different host-niches: those associated with the gastric glands (mucosal-dwelling larvae) and those either loosely associated with the mucosa or free-living in the lumen (lumen-dwelling larvae).

**Results:**

In this study excretory–secretory proteins from mucosal-dwelling and lumen-dwelling *T. circumcincta* fourth stage larvae were analysed using comparative 2-dimensional gel electrophoresis. A total of 17 proteins were identified as differentially expressed, with 14 proteins unique to, or enriched in, the excretory–secretory proteins of mucosal-dwelling larvae. One of the identified proteins, unique to mucosal-dwelling larvae, was a putative peroxiredoxin (*T. circumcincta* peroxiredoxin 1, Tci-Prx1). Peroxiredoxin orthologs from the trematode parasites *Schistosoma mansoni* and *Fasciola hepatica* have previously been shown to alternatively activate macrophages and play a key role in promoting parasite induced Th2 type immunity. Here we demonstrate that *Tci-Prx1* is expressed in all infective *T. circumcincta* life-stages and, when produced as a recombinant protein, has peroxidase activity, whereby hydrogen peroxide (H_2_O_2_) is reduced and detoxified. Furthermore, we use an *in vitro* macrophage stimulation assay to demonstrate that, unlike peroxiredoxins from trematode parasites *Schistosoma mansoni* and *Fasciola hepatica*, Tci-Prx1 is unable to alternatively activate murine macrophage cells.

**Conclusions:**

In this study, we identified differences in the excretory–secretory proteome of mucosal-dwelling and lumen-dwelling infective fourth-stage *T. circumcincta* larvae, and demonstrated the utility of this comparative proteomic approach to identify excretory–secretory proteins of potential importance for parasite survival and/or host immune modulation.

**Electronic supplementary material:**

The online version of this article (10.1186/s13071-019-3593-6) contains supplementary material, which is available to authorized users.

## Background

*Teladorsagia circumcincta* is an economically-important, widespread parasitic nematode of small ruminants in temperate regions. The life-cycle of *T. circumcincta* is a typical direct life-cycle employed by many species of trichostrongylid nematode, the parasitic phase of which is initiated by the exsheathed third larval stage (xL3) parasites in the abomasum of the small ruminant host [1]. Following entry of the xL3 parasites into the gastric pits and glands in the abomasum the larvae may fail to develop further or emerge into the lumen immediately after moulting to the fourth larval stage (L4) or, as a third alternative, develop within the mucosa, emerging into the lumen as late L4 or early adults [2]. Recently, we compared the transcriptomes of these latter two populations; fourth-stage *T. circumcincta* larvae which were of the same developmental stage but differed in their niche within the abomasum of the same host animal, being either mucosal-dwelling (MD) or lumen-dwelling (LD) [3]. That analysis identified a number of transcripts which were up-regulated in mucosal- compared to lumen-dwelling larvae, and which potentially encoded proteins, including immunomodulators, important for survival of the parasite within the mucosal environment where they are in intimate contact with the host’s tissues [3].

Host immunomodulation by many parasitic helminths is mediated through excretory/secretory (ES) products. Host immunomodulation is multifaceted and results in polarisation of the host immune response towards a Th2 phenotype; differentiation of macrophages towards an alternative (M2) phenotype; prevention of pro-inflammatory cytokine production by dendritic cells and the production of immunoregulatory molecules and induction of regulatory T cells (recently reviewed in [4]). This regulatory activity may not be specifically targeted at the host’s anti-helminth response but may have more general effects on the immune system. Therefore, helminth immune modulation has important implications, not only for the establishment of the helminth infection, but also for the outcomes of concomitant conditions or infections and the efficacy of immunodiagnostics and immunoprophylactic approaches (e.g. see [5]).

Our initial analyses of the induction of niche-specific expression of molecules by MD and LD L4 *T. circumcincta* took a differential transcriptomic approach [3]. The aim of the work described here is to determine whether that transcriptomic response is translated into the proteomic profiles of the ES material of MD and LD L4 *T. circumcincta* and to determine the putative functions of differentially expressed proteins in these two populations. One of these molecules, which we characterise here, is the anti-oxidant enzyme peroxiredoxin (Prx) which may be involved in the inactivation of host reactive oxygen species, and/or the induction of host Th2 responses through the induction of alternatively-activated macrophages as suggested for other helminth parasites [6, 7].

## Methods

### Generation of *T. circumcincta* L4 excretory/secretory (ES) proteins

Three 3-month old helminth-free Texel cross lambs were challenged *per os* with 150,000 infective *T. circumcincta* larvae (isolate MTci-2_CVL). After seven days, animals were euthanased and mucosal-dwelling (MD) and lumen-dwelling (LD) *T. circumcincta* L4 were collected as previously described [3]. Parasites isolated from each individual lamb were processed separately as follows: MD or LD L4 were washed three times in PBS before culturing in RPMI 1640 (Invitrogen, Carlsbad, CA, USA) containing 1% (v/v) D-glucose, 2 mM L-glutamine, 100 U/ml penicillin, 100 μg/ml streptomycin, 250 μg/ml gentamycin and 125 μg/ml amphotericin B, at 37 °C in 5% CO_2_. Culture supernatants were harvested after 24 h at which time the viability of the parasites was confirmed on the basis of structural integrity and motility. The culture supernatants were clarified by centrifugation, passed through 0.2 μm sterile filters and L4 ES products were subsequently concentrated approximately 40-fold using an Amicon Ultra-15 centrifugal filter with a 10 kDa cut-off (Sigma-Aldrich, St. Louis, MO, USA). Aliquots of ES products were stored at −80 °C prior to use.

### Comparative 2D-gel electrophoresis (2DE)

ES protein preparations from three paired ES samples generated from MD and LD L4 (MD L4 ES and LD L4 ES, respectively) from the same individual sheep were prepared for 2DE using a 2D Clean-Up kit (GE Healthcare Chicago, IL, USA) according to the manufacturer’s instructions and reconstituted in rehydration buffer (2% CHAPS, 7M urea, 0.3% DTT, 2M thiourea). Protein concentrations were then determined using a 2-D Quant kit (GE Healthcare, Chicago, IL, USA). For each ES preparation, a total of 23 µg protein was applied to a 7 cm Immobiline® Drystrip pH 3–10 NL (GE Healthcare, Chicago, IL, USA) and strips rehydrated overnight in SDS equilibration buffer (6 M urea, 75 mM Tris-HCl (pH 8.8), 29.3% glycerol, 2% SDS, 0.002% bromophenol blue) using an Immobiline DryStrip IPGbox (GE Healthcare, Chicago, IL, USA). Isoelectric focussing was then performed using a GE Healthcare Ettan™ IPGphor™ 3 Isoelectric Focusing System. The second-dimensional electrophoresis (SDS-PAGE) was performed by applying the strips to an ExcelGel 2-D Homogeneous 12.5% gel (GE Healthcare, Chicago, IL, USA). Following electrophoresis, gels were incubated with SimplyBlue™SafeStain (Invitrogen, Carlsbad, CA, USA) to visualise proteins. Gel images were captured using an ImageScanner III (GE Healthcare, Chicago, IL, USA) and images of MD and LD L4 ES were aligned using ImageMaster 2D Platinum software Version 7.05 (GE Healthcare, Chicago, IL, USA). A visual comparison of protein spots from MD *vs* LD L4 ES preparations was performed and differential spots were subsequently picked and analysed by LC-ESI-MS/MS as follows: excised 2-D spots were subjected to standard in-gel destaining, reduction, alkylation and trypsinolysis procedures [8]. Digests were transferred to low-protein-binding HPLC sample vials immediately prior to LC-ESI-MS/MS analysis. Liquid chromatography was performed using an Ultimate 3000 Nano-HPLC system (Dionex, Sunnyvale, CA, USA) comprising a WPS-3000 well-plate micro auto sampler, an FLM-3000 flow manager and column compartment, a UVD-3000 UV detector, an LPG-3600 dual-gradient micropump and an SRD-3600 solvent rack controlled by Chromeleon™ chromatography software (Dionex, Sunnyvale, CA, USA). A micro-pump flow rate of 246 µl min^−1^ was used in combination with a cap-flow splitter cartridge, affording a 1/82 flow split and a final flow rate of 3 µl min^−1^ through a 5 cm × 200 µm ID monolithic reversed phase column (Dionex, Sunnyvale, CA, USA) maintained at 50 °C. Samples of 4 µl were applied to the column by direct injection. Peptides were eluted by the application of a 15 min linear gradient from 8–45% solvent B (80% acetonitrile, 0.1% (v/v) formic acid) and directed through a 3 nl UV detector flow cell. LC was interfaced directly with a 3-D high capacity ion trap mass spectrometer (amaZon-ETD, Bruker Daltonics, Billerica, MA, USA) *via* a low-volume (50 µl min^−1^ maximum) stainless steel nebuliser (Agilent Technologies, Santa Clara, CA, USA, cat. no. G1946-20260) and ESI. Parameters for tandem MS analysis were based on those described previously [9].

### Database mining

The MS/MS data, formatted as Mascot Generic Format (mgf), was imported into ProteinScape™ V3.1 (Bruker Daltonics, Billerica, MA, USA) proteomics data analysis software for downstream mining of the NCBIprot database, using ‘Eukaryotes’ as the taxonomical search parameter. Database searches were conducted utilising the Mascot™ V2.5.1 (Matrix Science, Boston, MA, USA) search engine. Mascot search parameters were set in accordance with published guidelines [10] and to this end, fixed (carbamidomethyl “C”) and variable (oxidation “M” and deamidation “N, Q”) modifications were selected along with peptide (MS) and secondary fragmentation (MS/MS) mass tolerance values of 0.5 Da whilst allowing for a single 13C isotope. Using these criteria Mascot deemed scores over 68 to be significant. From the protein lists produced by Mascot the individual protein identifications scoring over 68 were inspected manually and considered significant only if (i) two peptides were matched for each protein; (ii) peptides were represented by a sequence coverage of > 5%; and (iii) each matched peptide contained an unbroken “b” or “y” ion series represented by a minimum of four contiguous amino acid residues.

### Stage-specific QPCR expression analysis of *Tci-Prx1*

Real-time quantitative polymerase chain reaction (QPCR) was performed using cDNA from *T. circumcincta* life stages which included: infective third stage larvae (iL3); carbon-dioxide exsheathed L3 (xL3); fourth-stage larvae (L4); and adult worms (all *T. circumcincta* life stage cDNA samples were kindly provided by Dr Thomas Tzelos, formerly of the Moredun Research Institute and details of their generation have previously been published in [3]). In addition, QPCR was also performed on cDNA from paired samples of *T. circumcincta* L4 lumen-dwelling (LD) worms and L4 mucosal-dwelling (MD) worms (cDNA was also generated in a previous study, described in [3]). Briefly, for each sample, total RNA was isolated from the nematodes using an RNeasy mini kit (Qiagen, Hilden, Germany), which included an on-column DNaseI treatment. First-strand cDNA was synthesized from 100 ng total RNA, using SuperScript III reverse transcriptase and oligo(dT)_20_ primer (Invitrogen, Carlsbad, CA, USA), according to the manufacturers protocol.

*Tci-Prx1* gene expression was compared in each *T. circumcincta* life stage using relative standard curve methodology. QPCR primers were designed using Primer3Plus [11] and specificity checked by PCR amplification and DNA sequencing of amplification fragments from L4 cDNA. Primer sequences are shown in Additional file 1: Table S1. For construction of standard curves, full length coding sequences for *Tci-Prx1* (accession number MG972995) and *Tci-β-tubulin* (accession number Z69258) were amplified from *T. circumcincta* L4 cDNA using Phusion proof-reading polymerase (Thermo Fisher Scientific, Waltham, MA, USA), and cloned into pJET1.2 (Thermo Fisher Scientific, Waltham, MA, USA). All sequences were verified by DNA sequencing, and used in QPCR experiments to construct standard curves from 10^8^–10^1^ copies of each gene. Twenty microliter QPCR reactions comprised 1× SYBR GreenER™ qPCR SuperMix Universal (Thermo Fisher Scientific, Waltham, MA, USA), 200 nM of forward and reverse primers, and cDNA derived from 1 ng total RNA for each sample. PCR reactions were performed on an Applied Biosystems 7500 Real Time PCR System; thermal cycling conditions were 50 °C for 2 min, 95 °C for 10 min, followed by 40 cycles at 95 °C for 30 s, 55 °C for 30 s and 72 °C for 30 s. Analysis of amplification profiles and melt curves was performed using Applied Biosystems 7500 software (v2.0.6). *Tci-Prx1* expression was normalized to housekeeping gene *Tci-β-tubulin* and expression reported relative to either iL3 expression levels, or LD L4 expression levels, depending on the experiment. QPCR experiments were performed in triplicate and included no template controls and no reverse transcription controls with each run.

### Expression and purification of endotoxin-free wild-type and mutant Tci-Prx1 proteins

The full-length predicted *Tci-Prx1* coding sequence was contained within a single contig in a collated *T. circumcincta* 28,143 contig transcriptomic database described previously [3]. The entire coding sequence was amplified from *T. circumcincta* L4 cDNA using primers that incorporated a 5′ *Nde*I restriction enzyme site and histidine-tag, and a 3′ *BamH*I restriction enzyme, site to allow directional cloning of the *Tci-Prx1* coding sequence into pET11a (primer sequences are shown in Additional file 1: Table S1). PCR amplification was completed on an Applied Biosystems 2720 thermal cycler with the following conditions: 98 °C for 30 s, followed by 30 cycles at 98 °C for 10 s, 56 °C for 10 s and 72 °C for 30 s. A mutant Tci-Prx1 (*mut Tci-Prx1*) coding sequence, lacking active site cysteines (C52A, C76A and C173A), was commercially synthesized (Eurofins Genomics, Ebersberg, Germany). Both wild-type wt and mut Tci-Prx1 coding sequences were digested with *Nde*I and *BamH*I and cloned into the corresponding sites of pET11a, and expression constructs were verified by DNA sequencing.

For production of LPS-free wt and mut Tci-Prx1, ClearColi BL21 (DE3) cells (Lucigen Corporation Middleton, WI, USA) were transformed with either wt or mut Tci-Prx1 expression constructs. Cells were grown in LB supplemented with 1% (w/v) glucose, 100 µg/ml ampicillin, and expression was induced by addition of 1mM isopropyl-d-1-thiogalactopyranoside (IPTG). Both wt and mut Tci-Prx1 were purified from soluble *E. coli* cell lysates, proteins were loaded onto HisTrap HP columns (GE Healthcare, Chicago, IL, USA) in binding buffer (20 mM Tris-HCl (pH 8.0), 0.5 M NaCl, 45 mM imidazole, 2 mM DTT) and eluted in binding buffer with a linear imidazole gradient.

Purified wt and mut Tci-Prx1 proteins were dialyzed overnight into PBS (137 mM NaCl, 2.7 mM KCl, 8.1 mM Na_2_HPO_4_, 1.5 mM KH_2_PO_4_) and residual endotoxin removed by 3 cycles of Triton-X114 phase separation, according to [12]. Prior to functional analysis, both wt and mut Tci-Prx1 were reduced with 2mM DTT for 60 min at room temperature. Excess DTT was removed by gel filtration using PD-10 desalting columns (GE Healthcare, Chicago, IL, USA) and eluted proteins were filter sterilized prior to use. Protein concentrations were determined by BCA assay (Thermo Fisher Scientific, Waltham, MA, USA) with BSA standards, and purity was confirmed by SDS-PAGE analysis. Endotoxin levels were determined using Pierce™ LAL Chromogenic Endotoxin Quantitation Kit (Thermo Fisher Scientific, Waltham, MA, USA) and murine macrophage like LPS-responsive reporter ELAM9 cells as described in [13].

### Peroxiredoxin activity assay

Reduction of peroxide (H_2_O_2_) was measured using the ferrous oxidation-xylenol orange (FOX) assay (Pierce^TM^ quantitative peroxide assay kit, Thermo Fisher Scientific, Waltham, MA, USA). Reactions were performed at 22 °C, and initiated by mixing 4 µM reduced wt Tci-Prx1 in PBS with 0.2 mM DTT, with 40 µM H_2_O_2_. After incubation for 30 min, reactions were quenched, according to manufacturer’s protocol. Negative control reactions were run in parallel using mut Tci-Prx1. Absorbance was measured at 562 nm on a microplate reader (BioTek Instruments, Inc., Winooski, VT, USA). Peroxide standards (4–120 µM) were included in each assay, and used to calculate the remaining quantities of H_2_O_2_. Replicates (*n* = 4) included wt Tci-Prx1 and mut Tci-Prx1 that were purified from 4 separate expression clones.

### Phylogenetic analysis

For phylogenetic analysis, protein sequences were aligned using MUSCLE [14] and ambiguously aligned positions were excluded by trimAL v1.2 [15]. Neighbour joining (NJ) phylogenetic trees, with 1000 bootstrap replicates, were constructed using MEGA v.6.06 [16].

### SDS-PAGE and Western blot analysis

Proteins were analysed by SDS-PAGE using the NuPAGE® electrophoresis system (Invitrogen, Carlsbad, CA, USA). Briefly, protein samples were prepared in NuPAGE LDS sample buffer with reducing agent and heated to 70 °C for 10 min prior to loading on NuPAGE 4–12% Bis-Tris gels. For analysis of wt and mut Tci-Prx1 under non-reducing conditions, reducing agent was omitted from the NuPAGE LDS sample buffer, and samples were not heated prior to loading onto gels. Gels were run in MES SDS running buffer and either stained with SimplyBlue^TM^ SafeStain (Invitrogen, Carlsbad, CA, USA) or prepared for use in Western blot experiments.

For Western blot analysis, purified rTci-Prx1; rTci-APY-1 and/or rSUMO-Tci-MEP-1 (500 ng of each) were resolved by SDS-PAGE under reducing conditions. mutTci-APY-1 and Tci-MEP-1 were produced and purified as previously reported by [17]. Proteins were transferred to nitrocellulose membranes by electroblotting, using 1X NuPAGE transfer buffer, according to the manufacturer’s instructions (Invitrogen, Carlsbad, CA, USA). After transfer, to prevent non-specific protein binding, membranes were blocked for 2 hrs at room temperature with 5% milk powder in TBST (20 mM Tris-HCl (pH 7.6), 150 mM NaCl, 0.1% (v/v) TWEEN20). Blots were washed three times with TBST, then probed with pooled sera from 7 helminth-naive six-month-old sheep at either day 0 (prior to *T. circumcincta* trickle infection) or day 112 (post-*T. circumcincta* trickle infection). Sera were generated in a previous study (described in [17]). Briefly, for the trickle infection each sheep was orally challenged with 2000 L3 *T. circumcincta*, three times per week for 4 weeks [17]. The first dose of infective larvae was administered to sheep 42 days after day 0 and the final dose was administered 70 days after day 0. Sheep sera were used at 1:3000 dilution in TBST for IgG blots and 1:500 dilution in TBST for IgA blots and incubated with membranes overnight at 4 °C. After washing three times in TBST, membranes were incubated with either: mouse monoclonal anti-goat/sheep IgG-Peroxidase antibody (Sigma-Aldrich, St. Louis, MO, USA) at 1:3000 dilution in TBST; or rabbit anti-sheep IgA HRP (Bio-Rad, Hercules, CA, USA) at 1:1000 dilution in TBST for 1 hour at room temperature. Membranes were washed three times with TBST and specifically bound antibodies were detected using SIGMAFAST^TM^ DAB with metal enhancer (Sigma-Aldrich, St. Louis, MO, USA).

### Endotoxin assay with recombinant Tci-Prx1

RAW264.7/ELAM-eGFP is a reporter cell line stably transfected with the E-selectin-eGFP (ELAM-eGFP) expression plasmid [13]. The ELAM promoter is induced by the activation of the NF-kappa B transcription factor, so when a pro-inflammatory stimulus such as LPS activates the ELAM promoter, the inducible expression of the green fluorescent protein (GFP) reporter gene is triggered. We incubated 10^6^ RAW264.7/ELAM-eGFP cells with 20 μg/ml of wt and mut Tci-Prx1 to determine if the proteins were contaminated with endotoxin. Controls consisted of cells incubated in media only (negative control) or with 20 μg/ml of LPS (0111:B4, Sigma-Aldrich, St. Louis, MO, USA) as a positive control. After a period of 4-h incubation, cells were harvested, washed twice with PBS and re-suspended in the dead cell stain Sytox Blue (Invitrogen, Carlsbad, CA, USA) prior to flow cytometry analysis. A minimum of 10,000 events were acquired using a MACSQuant® Analyzer 10 (Miltenyi Biotech, Bergisch Gladbach, Germany). Post-acquisition gating, including dead cell and doublet cell discrimination, and analysis were carried out using FlowJo vX for Windows 7.

### *In vitro* stimulation of macrophages with recombinant Tci-Prx1

RAW 264.7 murine macrophage-like cells [18] were cultured in DMEM (Sigma-Aldrich, St. Louis, MO, USA) containing 10% fetal calf serum (FCS), 200 U/ml penicillin and 200 µg/ml streptomycin at 37 °C, 5% CO_2_. Cell cultures were split once 1:5 prior to stimulation. For stimulation assays, 5 × 10^4^ cells were plated in duplicate into 12-well flat bottomed tissue culture plate (Nalge Nunc International Corp., Roskilde, Denmark), with fresh culture media containing either: 20 ng/ml recombinant murine IL-4 (PeproTech, London, UK); 100 ng/ml recombinant murine IFN-γ (PeproTech, London, UK); 20 µg/ml wt Tci-Prx1; or 20 µg/ml mut Tci-Prx1. For unstimulated (negative control), cells were incubated with culture media only. After 24 h of stimulation, media was harvested and cells washed twice with PBS and lysed in RLT buffer with β-mercaptoethanol (Qiagen, Hilden, Germany). RNA was isolated using an RNeasy mini kit (Qiagen, Hilden, Germany), which included an on-column DNaseI treatment. First-strand cDNA was synthesized from 100 ng total RNA, using SuperScript III reverse transcriptase and oligo(dT)_20_ primer (Thermo Fisher Scientific, Waltham, MA, USA), according to the manufacturers protocol. QPCR was used to quantify gene markers of M1 activation (IL-6, iNOS, and TNF-α) and M2 activation (arginase and mannose receptor 1) in RAW 264.7 cells across all treatments. QPCR primers were designed using Primer3Plus [11], and specificity checked by PCR amplification and DNA sequencing of amplification fragments from RAW 264.7 cell cDNA. Primer sequences are shown in Additional file 1: Table S1. For construction of standard curves, coding regions for: β-actin (NM_007393); arginase (NM_007482); IL-6 (NM_031168); iNOS (NM_010927); TNF-α (NM_013693) and MRC1 (NM_008625) were amplified from either RAW264.7 cell cDNA, or mouse gDNA using Phusion proof-reading polymerase (Thermo Fisher Scientific, Waltham, MA, USA) and cloned into pJET1.2 (Thermo Fisher Scientific, Waltham, MA, USA). All sequences were verified by DNA sequencing, and used in QPCR experiments to construct standard curves from 10^1^–10^8^ copies of each gene. QPCR reactions were performed as described previously (see above), with the following changes: twenty microliter QPCR reactions comprised 1× PowerUp SYBR green master mix (Thermo Fisher Scientific, Waltham, MA, USA), 500 nM of forward and reverse primers, and cDNA derived from 1 ng total RNA for each sample. PCR reactions were performed on an Applied Biosystems 7500 Real Time PCR System; thermal cycling conditions were 50 °C for 2 min, 95 °C for 2 min, followed by 40 cycles at 95 °C for 15 s, 50 °C for 15 s, and 72 °C for 1 min. Gene expression was normalized to housekeeping gene *β-actin* and expression reported relative to media only (negative control) expression levels, QPCR experiments were performed in triplicate and included no template controls and no reverse transcription controls with each run.

### Statistical analysis

Peroxiredoxin activity assay data and QPCR data were analysed using GraphPad Prism version 8.0.0 for Windows (GraphPad Software, La Jolla California USA). Datasets were analysed using a Student’s t-test. *P* values of < 0.05 were considered significant.

## Results

### Niche-specific excretome/secretome of lumen- and mucosal-dwelling fourth-stage *T. circumcincta* larvae

To identify niche-specific excretory–secretory (ES) proteins in lumen-dwelling (LD) and mucosal-dwelling (MD) fourth-stage *T. circumcincta* larvae (L4), populations of both mucosal and larval parasites were collected, from abomasa of sheep, 7 days following an oral challenge with L3 parasites and processed as 2 separate populations. The recovered L4 parasites were cultured in serum-free conditions, according to methods described by Geldhof et al. [19]. Using these culture conditions, *T. circumcincta* L4 larvae remained viable for at least 48 h on the basis of structural integrity and motility. For analysis of *T. circumcincta* ES proteins, culture supernatants were collected after 24 h and concentrated for proteomic identification of the L4 ES proteins.

**Fig. 1:**
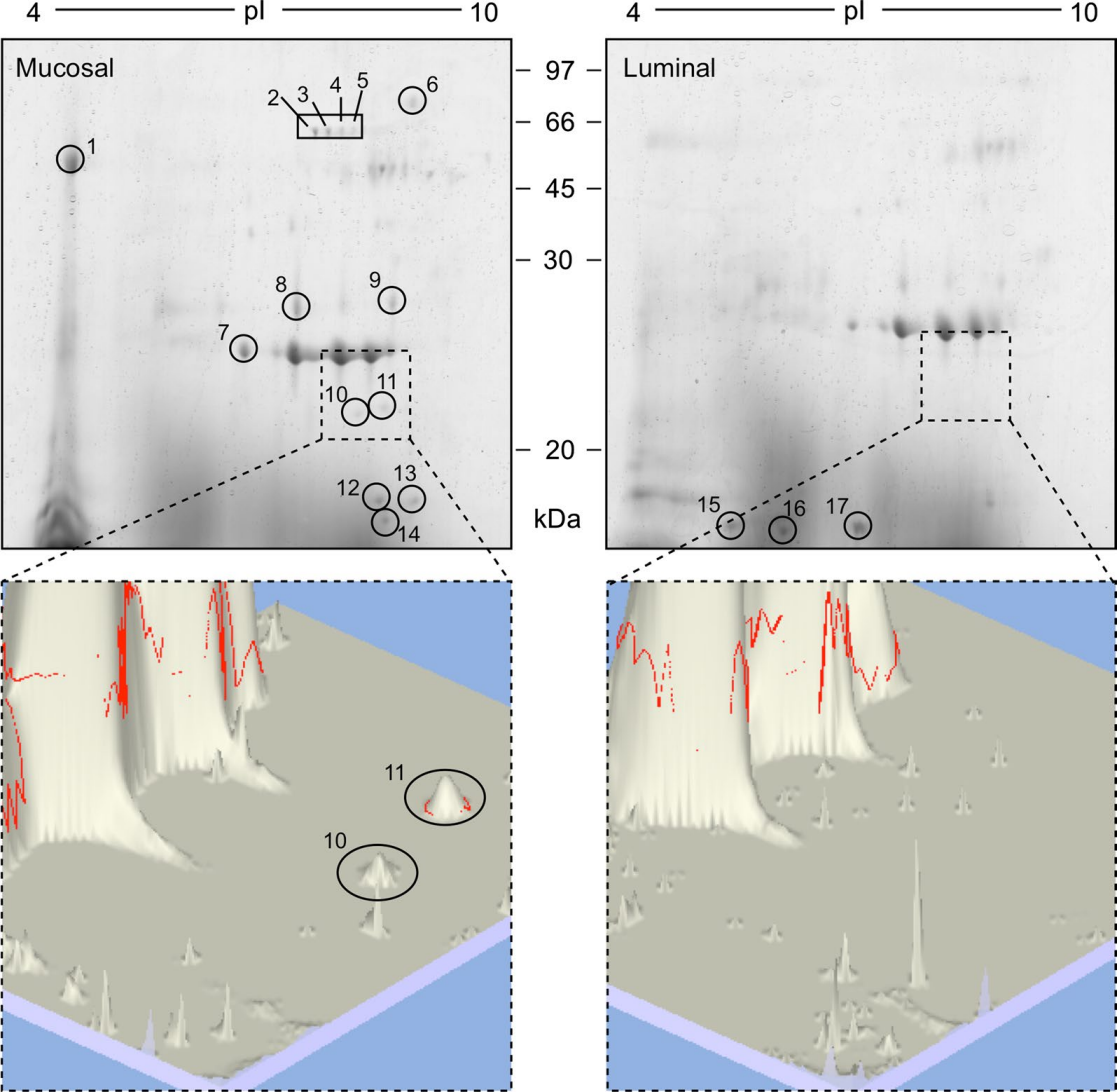
2D gels of excretory–secretory (ES) proteins of fourth-stage *T. circumcincta* larvae. ES proteins (23 µg of each) were separated by 2D gel electrophoresis and stained with SimplyBlue^TM^ SafeStain (Invitrogen). Protein spots upregulated in either *T. circumcincta* L4 mucosal-dwelling ES proteins or *T. circumcincta* L4 lumen-dwelling ES proteins are numbered and their protein identifications are shown in Table 1. The two lower panels show 3-dimensional images generated from the areas outlined in the dashed boxes on the gels with the relevant spots circled

L4 ES proteins were compared by 2D gel electrophoresis (2DE) and Coomassie blue staining (Fig. 1). A total of 17 spots were identified as differentially expressed between LD and MD parasites; with 11 spots unique to the ES of MD parasites (spots 1–6 and spots 10–14; Fig. 1 Mucosal); 3 spots enriched in the ES of MD parasites compared to LD parasites (spots 7–9; Fig. 1 Mucosal); and 3 spots unique to the ES of LD parasites (spots 15–17; Fig. 1 Luminal).

MS analysis of excised ES protein spots identified 9 *T. circumcincta* proteins (Table 1). In addition, because of the close association of MD parasites with the host mucosa, some *Ovis aries* (host) proteins were also identified in the ES protein fractions from MD parasites (spots 2–5 and 10–13; Table 1).

**Table 1:** Fourth-stage *T. circumcincta* ES protein identities. Spot numbers refer to those in Fig. 1. NCBI protein ID shows top-hit from NCBI non-redundant protein sequences (nr) database; single protein identities are shown when the same peptides match protein isoforms (with the longest isoform shown); multiple protein identities are shown when different proteins are identified within a single spot

Spot	NCBI protein ID	Species	Protein accession	MW	MOWSE score	No. of peptides (% coverage)
1	Hypothetical protein TELCIR_08116	*Teladorsagia circumcincta*	PIO70037	13.1	496	6 (38.1)
	ShK domain protein	*Teladorsagia circumcincta*	PIO55996	44.7	82	8 (20.2)
2	Serum albumin precursor	*Ovis aries*	NP_001009376	69.1	615	34 (56.8)
3	Serum albumin precursor	*Ovis aries*	NP_001009376	69.1	745	30 (50.9)
4	Serum albumin precursor	*Ovis aries*	NP_001009376	69.1	610	30 (52.6)
5	Serum albumin precursor	*Ovis aries*	NP_001009376	69.1	517	26 (43.8)
6	Hypothetical protein TELCIR_10830	*Teladorsagia circumcincta*	PIO67421	24.3	90	4 (19.7)
7	Secreted cathepsin F	*Teladorsagia circumcincta*	ABA01328	41.1	338	11 (31)
8	Activation-associated secretory protein	*Teladorsagia circumcincta*	CBJ15404	26.5	577	13 (45.8)
9	Activation-associated secretory protein	*Teladorsagia circumcincta*	CBJ15404	26.5	413	15 (47.5)
10	Gastrokine-1 precursor	*Ovis aries*	NP_001093100	20.4	189	8 (33)
11	Gastrokine-1 precursor	*Ovis aries*	NP_001093100	20.4	610	10 (37.3)
	Tci-Prx1	*Teladorsagia circumcincta*	MG972995	22.1	231	5 (26.6)
12	Galectin 15 (LGALS15)	*Ovis aries*	ABS30837	15.4	192	4 (33.6)
13	Galectin 15 (LGALS15)	*Ovis aries*	ABS30837	15.4	279	6 (47.4)
14	No ID					
15	Hypothetical protein TELCIR_00024	*Teladorsagia circumcincta*	PIO77845	17.1	241	2 (17.2)
16	Hypothetical protein TELCIR_00024	*Teladorsagia circumcincta*	PIO77845	17.1	237	4 (31.1)
	Transthyretin precursor, partial	*Teladorsagia circumcincta*	PIO54996	10.4	173	3 (34.8)
	Hydroxyisourate hydrolase	*Teladorsagia circumcincta*	PIO60950	15.4	173	3 (23.7)
17	No ID					

Proteomic analysis of *T. circumcincta* ES proteins confirmed the presence of a previously described *T. circumcincta* L4 specific secreted cathepsin F (spot 7) [20] and a *T. circumcincta* L4 specific activation-associated secretory protein (spot 8 and 9) [21]. Identification of additional *T. circumcincta* ES proteins included: peroxiredoxin 1 (Tci-Prx1; spot 11) unique to MD parasites; transthyretin/ hydroxyisourate hydrolase (spot 16) unique to LD parasites; and hypothetical proteins of unknown function (spots 1, 6, 15 and 16). To assign biological function to those *T. circumcincta* hypothetical proteins of unknown function (Table 1), 3D structural models were generated using I-TASSER, and best-fit models were compared against Protein Data Bank (PDB) proteins [22]. Using this approach, *T. circumcincta* hypothetical proteins TELCIR_08116 (spot 1), TELCIR_10830 (spot 6), and TELCIR_00024 (spot 15 and 16) were shown to have high levels of structural similarity to CAP-domain proteins from the human hookworm *Necator americanus* [23] with TM scores of 0.874, 0.873 and 0.643 respectively (Table 2). Additionally, *T. circumcincta* ShK domain protein (spot 1) had very high levels of structural similarity (TM score 0.902) to human meprin β metalloproteinase, a membrane anchored protease that sheds membrane-bound cytokines and growth factors [24] (Table 2).

**Table 2:** I-TASSER structure/function predictions of *T. circumcincta* ES hypothetical proteins. Spot numbers refer to those in Fig. 1. 3D structural models of *T. circumcincta* hypothetical proteins were generated by I-TASSER [22] and compared to Protein Bata Bank (PDB) proteins. Template modelling score (TM-score) shows the structural similarity between the I-TASSER predicted protein structure model and PDB protein. TM-score = 1 indicates a perfect match between two structures; TM-score > 0.5 indicates a model of correct topology

Spot	NCBI protein ID	Protein accession	Protein Data Bank (PDB) top hit [PDB ID]; protein description	TM-score	Reference
1	Hypothetical protein TELCIR_08116	PIO70037	[3NT8]; *Necator americanus* ASP-1 (Na-ASP-1)	0.874	[23]
	ShK domain protein	PIO55996	[4GWM]; Human promeprin beta	0.902	[24]
6	Hypothetical protein TELCIR_10830	PIO67421	[3NT8]; *Necator americanus* ASP-1 (Na-ASP-1)	0.873	[23]
15 and 16	Hypothetical protein TELCIR_00024	PIO77845	[3NT8]; *Necator americanus* ASP-1 (Na-ASP-1)	0.643	[23]

To discriminate between Tci-Prx1 and *O. aries* (host) peroxiredoxins (which have amino acid sequence identities between 69–74% with Tci-Prx1), all sequenced peptides from spot 11 (Fig. 1) were mapped to amino acid sequence alignments of Tci-Prx1 and *O. aries* peroxiredoxins 1, 2 and 4 (OaPrx1, OaPrx2 and OaPrx4). All sequenced peptides from spot 11 (Fig. 1) were unique to Tci-Prx1 (Additional file 2: Figure S1).

### Infective stage *T. circumcincta* larvae have upregulated transcription of *Tci-Prx1*

**Fig. 2:**
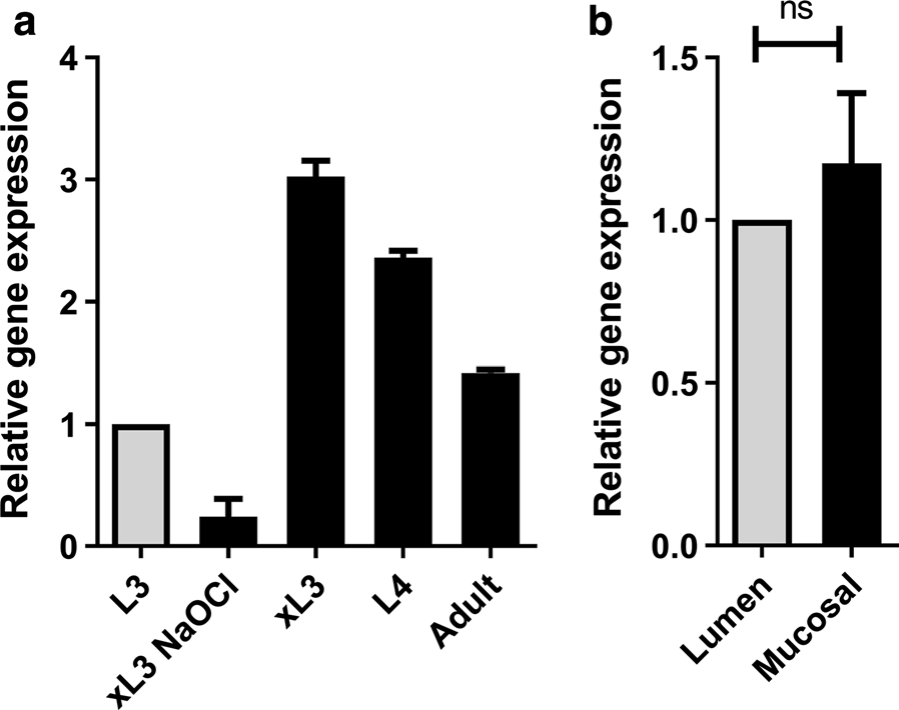
Stage specific expression of *Tci-Prx1*. **a** qPCR gene expression analysis of *Tci-Prx1* mRNA in *T. circumcincta* stage 3 larvae (L3); sodium hypochlorite exsheathed *T. circumcincta* L3 (xL3 NaOCl); carbon dioxide exsheathed *T. circumcincta* L3 (xL3); fourth-stage *T. circumcincta* larvae (L4); and adult *T. circumcincta* worms (Adult). **b** qPCR gene expression analysis of *Tci-Prx1* mRNA in *T. circumcincta* L4 lumen-dwelling (Lumen) worms and *T. circumcincta* L4 mucosal-dwelling (Mucosal) worms. Each value is the mean ± SEM, *n* = 3. There were no significant differences between lumen and mucosal expression levels (t-test, *t* = 0.8106, *df* = 2, *P* = 0.724). All data were normalized to *Tci-β-tubulin* and shown relative to either L3 expression (**a**) or Lumen expression (**b**)

QPCR gene expression analyses of *T. circumcincta* third-stage larvae (L3), CO_2_ exsheathed L3 (CO_2_ xL3), fourth-stage larvae (L4) and adult worms showed that the *Tci-Prx1* mRNA transcript is upregulated in host-associated stages relative to free-living L3 expression levels (Fig. 2a). *Tci-Prx1* expression was increased 3-fold in CO_2_ xL3, 2.4-fold in L4 and 1.4-fold in adults relative to L3 expression levels (Fig. 2a). Interestingly, chemical exsheathment of L3 parasites using sodium hypochlorite solution resulted in a 4.1-fold reduction in *Tci-Prx1* expression relative to L3 expression levels (Fig. 2a), suggesting that exsheathment with CO_2_ may better represent conditions within the rumen associated with natural exsheathment compared to chemical “de-sheathment” with sodium hypochlorite. PCR analysis using cDNA from *T. circumcincta* L4 mucosal-dwelling parasites and *T. circumcincta* L4 lumen-dwelling parasites showed no statistically-significant differential niche-specific expression at the transcript level of *Tci-Prx1* (t-test, *t* = 0.8106, *df* = 2, *P* = 0.724) (Fig. 2b).

### Parasite Tci-Prx1 and its relationship with host peroxiredoxins

Tci-Prx1 shares high sequence similarity with AhpC-Prx1 subfamily (typical 2-cys) peroxiredoxins from other parasitic nematodes, including peroxiredoxins from *Ostertagia ostertagi*, *Haemonchus contortus* and *Ancylostoma ceylanicum*, with 99%, 95% and 90% amino acid identity to Tci-Prx1, respectively (Additional file 3: Figure S2). High levels of sequence identity were also observed between Tci-Prx1 and host peroxiredoxins OaPrx1, OaPrx2, and OaPrx4, with 73%, 74% and 73% amino acid identity, respectively. Sequence analysis demonstrated that Tci-Prx1 shares amino acid residues conserved across all peroxiredoxin families, including a peroxidatic cysteine residue (cys52) and resolving cysteine residue (cys173) which are essential for peroxidase activity (Additional file 3: Figure S2).

**Fig. 3:**
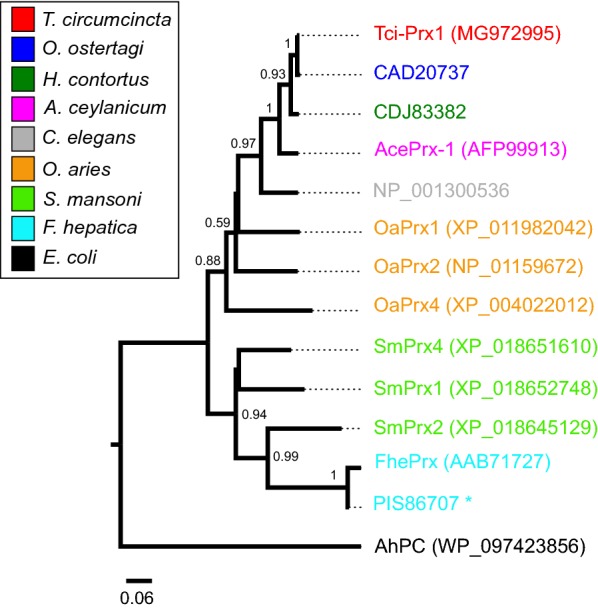
Phylogenetic analysis of Tci-Prx1 and related peroxiredoxins. Neighbour joining phylogenetic reconstruction of Tci-Prx1 (red) and selected AhpC-Prx1 subfamily (typical 2-cys) peroxiredoxins from helminths and vertebrate species (as indicated in figure). The tree is rooted using alkyl hydroperoxide reductase C (AhPC) from *Escherichia coli*. The reliability of the tree was determined using 1000 bootstrap replications, bootstrap values > 50% are shown. All sequences are available from NCBI using the accession numbers in the phylogenetic tree. Where protein isoforms exist, the longest isoform is included in the alignment. The scale-bar represents 0.06 substitutions per amino acid site. Peroxiredoxins with a signal peptide are indicated (*)

The phylogenetic relationship of Tci-Prx1 with other peroxiredoxins was demonstrated by comparison with AhpC-Prx1 subfamily (typical 2-cys) peroxiredoxins from representative helminth species and peroxiredoxins from the *T. circumcincta* mammalian host *O. aries* (Fig. 3). Phylogenetic reconstruction placed Tci-Prx1 in a well-supported clade containing typical 2-cys peroxiredoxins from nematodes and three closely related typical 2-cys peroxiredoxins from its sheep host (OaPrx1, OaPrx2, and OaPrx4) (Fig. 3). All nematode and sheep typical 2-cys peroxiredoxins formed a group distinct from trematode typical 2-cys peroxiredoxins (Fig. 3). In terms of phylogeny and sequence identity, Tci-Prx1 is closely related to host *O. aries* peroxiredoxins (73–74% sequence identity). In comparison, trematode peroxiredoxins from *F. hepatica* and *S. mansoni* are more distantly related to mammalian *O. aries* peroxiredoxins (62–68% sequence identity), suggesting that these trematode peroxiredoxins may have important differences in structure and/or function.

### Antibody responses to rTci-Prx1 in *T. circumcincta* infected lambs

**Fig. 4:**
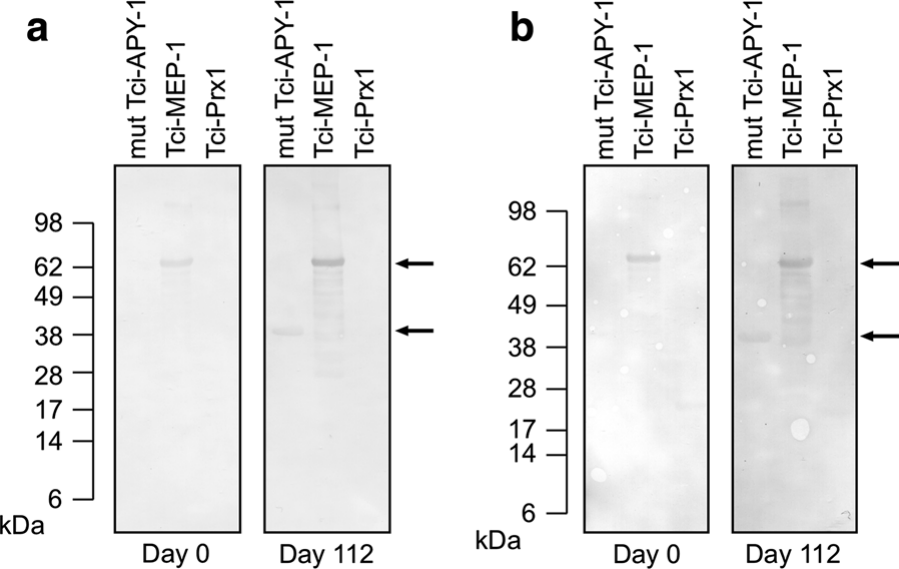
Immune recognition of rTci-Prx1 using sera from *T. circumcincta* trickle-infected sheep. Purified recombinant Tci-Prx1 and other recombinant *T. circumcincta* ES proteins including Tci-MEP-1 and mut Tci-APY-1 (500 ng of each) were resolved by SDS-PAGE and proteins transferred to nitrocellulose membranes. Blots were probed with pooled sera from 7 helminth-naïve six-month-old sheep at either day 0 (prior to *T. circumcincta* trickle infection); or day 112 (post *T. circumcincta* trickle infection). Blots shown in **a** were probed with anti-goat/sheep IgG-HRP secondary antibodies. Blots shown in **b** were probed with anti-sheep IgA-HRP secondary antibodies. Specifically bound antibodies were visualised using DAB staining. Arrows indicate presence of either Tci-MEP-1 (57 kDa) or mut Tci-APY-1 (38 kDa)

Recombinant Tci-Prx1 was not bound by either IgG or IgA antibodies present in serum from 6-month-old helminth-naïve sheep (Fig. 4a, b; Day 0) or in serum from the same lambs after a 4-week oral *T. circumcincta* trickle infection (Fig. 4a,b; Day 112). In comparison, following *T. circumcincta* trickle infection, recombinant versions of two other *T. circumcincta* L4 ES proteins (Tci-MEP-1 and Tci-APY-1) were both bound by serum IgG antibodies (Fig. 4a; Day 112) and serum IgA antibodies (Fig. 4b; Day 112).

### rTci-Prx1 is an active cysteine-dependent peroxidase

**Fig. 5:**
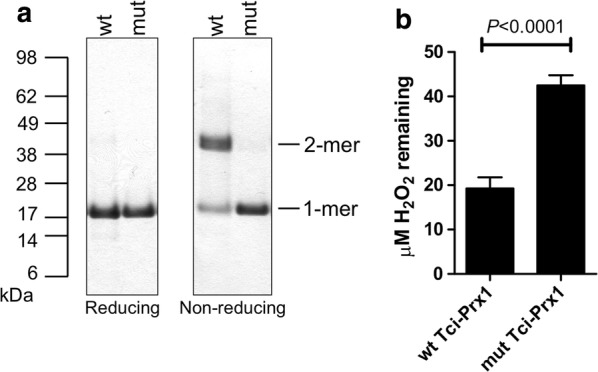
Biochemical analysis of wild-type and mutant Tci-Prx1. **a** SDS-PAGE analysis of purified wild-type (wt) and mutant (mut) Tci-Prx1 (5 µg of each protein/lane) under reducing and non-reducing conditions. Presence of monomer (1-mer, 23.3 kDa) and dimer (2-mer, 46.6 kDa) is indicated. **b** Peroxidase activity of wt and mut Tci-Prx1. Each reaction contained 40 µM of H_2_O_2_ and either wt Tci-Prx1 or mut Tci-Prx1, and diminishment of peroxide was monitored by FOX assay. Each value is the mean ± SEM, *n* = 5. Significant differences between wt Tci-Prx1 and mut Tci-Prx1 are shown (t-test, *t* = 6.768, *df* = 4, *P* < 0.001)

Purified wild-type (wt) and mutant (mut) recombinant Tci-Prx1 migrated as monomers with an apparent molecular weight of *c.*20 kDa when analysed by reducing SDS-PAGE, in agreement with their predicted molecular weight (Fig. 5a). Under non-reducing conditions, wt Tci-Prx1 migrated principally as a dimer, with an apparent molecular weight of 40 kDa. Under all conditions tested the triple cysteine Tci-Prx1 mutant (C52A; C76A and C173A) was unable to dimerize and migrated as a monomer with an apparent molecular weight of 20 kDa (Fig. 5a).

The enzyme activity of purified recombinant Tci-Prx1 was determined using an *in vitro* assay that measures the Tci-Prx1-catalysed diminishment of hydrogen peroxide (H_2_O_2_) in solution. The specific activity of wt Tci-Prx1 was calculated as 8.2 ± 0.944 nmol H_2_O_2_ reduced min^−1^ mg^−1^ (*n* = 5) (Fig. 5b). Mut Tci-Prx1, which lacks the peroxidatic cysteine residue (cys52) and resolving cysteine residue (cys173) had no detectable enzyme activity (Fig. 5b).

### Purified rTci-Prx1 does not activate RAW 264.7 macrophage cells

Secreted 2-Cys peroxiredoxins from parasitic trematodes *Fasciola hepatica* (FhPrx1) and *Schistosoma mansoni* (SmPrx1) are dual function enzymes that inactivate reactive oxygen species (ROS), and also induce the production of alternatively activated macrophages (M2 macrophages) in mouse models [6, 7]. Thus, we performed experiments to test whether Tci-Prx1 could also induce production of M2 macrophages.

**Fig. 6:**
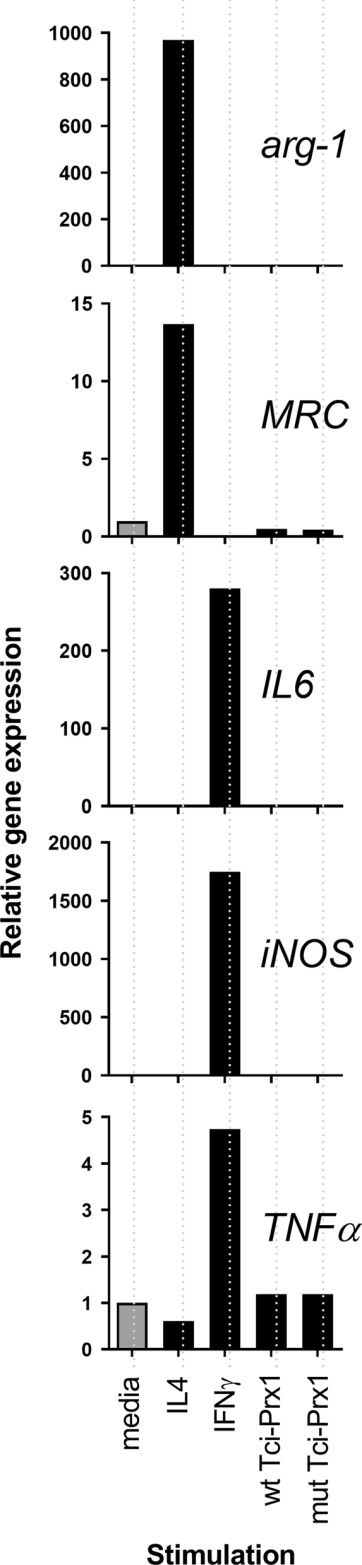
*In vitro* stimulation of macrophages with recombinant Tci-Prx1. qPCR gene expression analysis of markers of M1 activation (IL-6, iNOS, TNF-α) and M2 activation (arginase and MRC-1) in RAW 264.7 cells following stimulation with IL4 (20 ng/ml), IFN-γ (100 ng/ml), wt Tci-Prx1 (20 µg/ml) and mut Tci-Prx1 (20 µg/ml). Unstimulated negative control cells (-ve) were run in parallel and incubated with culture media only. All data were normalized to *β-actin* and shown relative to media only (-ve control) expression levels

Both purified recombinant wt Tci-Prx1 and mut Tci-Prx1 had low levels of endotoxin (< 0.1 EU/ml) and consequently did not activate murine macrophage-like LPS-responsive reporter cell line RAW264.7/ELAM-eGFP [13] (Additional file 4: Figure S3). For macrophage M1/M2 polarization experiments, murine macrophage-like RAW 264.7 cells were stimulated for 24 h with either wt Tci-Prx1 or mut Tci-Prx1. Cells were also stimulated with recombinant murine interferon gamma (rIFN-γ) or recombinant murine interleukin-4 (rIL4) as controls to induce M1 or M2 polarisation, respectively [25]. Relative to negative control cells (no stimulation), wt Tci-Prx1 or mut Tci-Prx1 did not induce transcription of markers of either M1 polarization (IL-6, iNOS, and TNF-α) or M2 polarization (arginase and mannose receptor 1) (Fig. 6). In contrast, relative to unstimulated controls, rIFN-γ resulted in strong induction of genetic markers of M1 activation (IL-6, iNOS, and TNF-α); while rIL4 resulted in strong induction of genetic markers of M2 activation (arginase and mannose receptor 1) (Fig. 6).

## Discussion

Here, we have demonstrated the niche-specific expression of a suite of ES proteins from the fourth larval stages of *T. circumcincta* and demonstrated the potential of one of these proteins, Tci-Prx1, which is enriched in the ES of mucosal-dwelling as opposed to lumen-dwelling worms, to modulate the host immune response by elimination of host-derived reactive oxygen species which may be released from a range of immune effector cells to act locally to harm the parasites [26].

Previous proteomic studies of *T. circumcincta* L4 ES using 1- and 2-dimensional SDS-PAGE analyses have demonstrated the dominance of metallo- and cysteine proteinases in the L4 ES (in particular Tci-CF-1) and also a large complement of SCP/TAPS proteins (ASPs in particular) [21, 27] but neither of these studies identified Tci-Prx1 in the ES material. A more recent analysis of *T. circumcincta* L4 ES demonstrated the presence of Prx-like proteins in ES enriched for extracellular vesicles (EVs) and also ES depleted of EVs, suggesting that the enzyme is present in solution in ES as well as being encapsulated in EVs [28]. Similarly, peroxiredoxin has been reported in ES from the nematodes *Heligmosomoides polygyrus* [29] and associated with EVs from *Brugia malayi* [30]; in addition peroxiredoxin is present in EVs from the trematodes *F. hepatica* and *Echinostoma caproni* [31, 32]. These combined studies suggest that peroxiredoxin is an important conserved secretory molecule across phylogenetically diverse parasitic helminths.

Differential transcriptomic analyses of MD and LD *T. circumcincta* L4s [3] also demonstrated the increased expression of transcripts encoding SCP/TAPS proteins, metalloproteinases and Shk-domain containing proteins in MD larvae and each of these protein classes were represented in the ES proteins identified as being unique to, or upregulated in, MD L4 ES in the work described herein (Tables 1 and 2). The previous differential transcriptomic analyses of MD and LD *T. circumcincta* L4s [3] did not identify the transcript encoding Tci-Prx1 as being differentially expressed between MD and LD larvae and this is reflected here at the transcriptomic level (Fig. 2b). It is clear, however, from 2D gel analyses (represented in Fig. 1) that Tci-Prx1 protein is present in the ES of MD larvae in particular, although we cannot discount its presence at low concentration, and thus undetectable levels, in LD ES. This apparent discord between transcriptomic analyses and proteomic analyses is well-known, with transcript levels not always providing a reliable proxy for protein abundance due to regulatory processes at the post-transcriptional level [33]. Comparative proteomic analysis presented in Fig. 1 demonstrates that Tci-Prx1 protein is detectable in MD *T. circumcincta* parasites. In addition, at a gene transcription level, *Tci-Prx1* is associated with the transition to parasitism and is particularly associated with those stages of the parasite (exsheathed L3 and L4) most intimately associated with the host mucosa (Fig. 2a).

During establishment of an infection, *T. circumcincta* L3 larvae pass through the rumen (a high CO_2_ environment), we hypothesize that ensheathed L3 larvae sense elevated levels of CO_2_ which triggers exsheathment and upregulation of a suite of genes encoding ES proteins that are essential for establishment of infection in the abomasum. In support of this CO_2_ sensing hypothesis, we have previously shown that *T. circumcincta* ES genes encoding activation associated protein-2 (Tci-ASP-2) and excretory secretory 14kDa (Tci-ES14-1) are both upregulated following CO_2_ exsheathment [3]. Here we have demonstrated that expression of *Tci-Prx1* is upregulated 3-fold in CO_2_ exsheathed *T. circumcincta* L3, relative to free-living *T. circumcincta* L3, which probably reflects remapping of gene expression during the transition from free-living to parasitic life-stages.

Recombinant wild-type Tci-Prx1 dimerised appropriately to permit *in vitro* analysis of its potential role(s) in host immunomodulation. Peroxidase activity, and thus the ability to mitigate the effects of host-derived reactive oxygen species, was demonstrated for the recombinant wt Tci-Prx1 (Fig. 5). In comparison to wt Tci-Prx1 peroxidase activity levels the *in vitro* peroxidase activity of other typical 2-cys peroxiredoxins from *F. hepatica* (1200 nmol min^−1^ mg^−1^) [34] and *A. ceylanicum* (1640 nmol min^−1^ mg^−1^) [35] was 146-fold higher and 200-fold higher, respectively. It is likely that the relatively low levels of Tci-Prx1 peroxidase activity is due to the lack of an appropriate physiological reductant in the *in vitro* assay [34, 36], and therefore reflects Tci-Prx1 peroxidase activity under partial or single turnover conditions [37].

In terms of other interactions with the host immune system, no Tci-Prx1-specific serum IgG or serum IgA was present in the sera of lambs which had been exposed to an extended trickle infection of *T. circumcincta*, though antigen-specific IgG and IgA against ES proteins, Tci-MEP-1 and Tci-APY-1, was present in these lambs. This suggests that Tci-Prx1 excreted or secreted from the developing larvae is not immunogenic in spite of being in released by parasites in close association with the host mucosa. However, it is also possible that the recombinant form of Tci-Prx1 may lack conformational B cell epitopes or post-translational modifications present on the native protein which are targeted by the parasite-induced antibody response.

It is widely accepted that parasitic helminths, regardless of the parasite or site of infection, typically induce strong type-2-cell-mediated immune responses that either control infection or mediate tolerance to the infection [38]. During parasitic helminth infections, alternatively activated macrophages (AAMϕ or M2) play a key role in promoting Th2 immune responses, while supressing pro-inflammatory Th1 responses [39]. In parasitic trematodes *S. mansoni* and *F. hepatica,* peroxiredoxins secreted in ES inactivate ROS, and also act as pathogen-associated molecular pattern molecules (PAMPs) where they alternatively activate macrophages, using a mechanism that is independent of antioxidant function [6, 7, 26, 40]. Both size-fractionated *F. hepatica* ES containing peroxiredoxin and purified recombinant *F. hepatica* peroxiredoxin (FhePrx) induce expression of arginase-1 in macrophages recruited to the injection site in BALB/c mice, and also induce expression of arginase-1 in *in vitro* cultured 264.7 RAW macrophages, indicative of macrophage switching to an alternative activated phenotype [6]. Consequently, we tested whether purified rTci-Prx1 is able to act as a PAMP, and drive alternative activation of *in vitro* cultured murine 264.7 RAW macrophage cells. In comparison to trematode peroxiredoxins, Tci-Prx1 was not able to induce cultured 264.7 RAW macrophage cells to an (M2) alternative activated phenotype, and resulted in no induction of arginase-1 (arg-1) or mannose receptor (MRC) gene expression. Thus, in comparison to previously described trematode peroxiredoxins [6, 7], rTci-Prx1 has anti-oxidant activity, but does not function as a PAMP and drive alternative activation of *in vitro* cultured murine macrophage cells. Although rTci-Prx1 did not activate murine macrophage cells, it is possible that rTci-Prx1 may not fully replicate the function of the native *T. circumcincta* Prx1, therefore future studies will be directed at molecular characterisation of native Tci-Prx1 from ES material.

Peroxiredoxins are generally well conserved across helminth parasites, with nematode and trematode ES peroxiredoxins sharing between 66–69% amino acid sequence identity. However, as demonstrated in this study, in terms of PAMP activity, and the ability to alternatively activate macrophage function, there are functional differences between Tci-Prx1 and previously characterised trematode peroxiredoxins from *S. mansoni* and *F. hepatica*. Further characterization of these structure/function differences will pave the way to further understanding peroxiredoxins in the context of parasite/host interactions, and their ability to modulate host immune responses.

## Conclusions

This study has identified differences in the excretory–secretory proteome of mucosal-dwelling and lumen-dwelling infective fourth-stage *T. circumcincta* larvae. We propose that excretory–secretory proteins produced by mucosal-dwelling larvae are important for parasite survival and/or modulation of the host immune response in the mucosal environment. In support of this, one of the identified excretory–secretory proteins, present in mucosal-dwelling *T. circumcincta* larvae, is a putative peroxiredoxin (*T. circumcincta* peroxiredoxin 1, Tci-Prx1). Peroxiredoxin orthologs from the trematode parasites *F. hepatica* and *S. mansoni* have previously been shown to be dual-function enzymes, with anti-oxidant enzyme activity, and also induce the production of alternatively activated macrophages. We demonstrate that although there is high sequence similarity between Tci-Prx1 and Prx proteins from trematodes *F. hepatica and S. mansoni*, the encoded proteins are functionally different. When expressed as a recombinant protein, Tci-Prx1 has anti-oxidant activity, but in an in vitro assay is unable to alternatively activated macrophages. Important functional differences between Tci-Prx1 and trematode Prxs likely reflect different parasite life-history strategies.

## Additional files

**Additional file 1: Table S1.** Primers for QPCR and Tci-Prx-1 gene expression construct.

**Additional file 2: Figure S1.** Alignment of Tci-Prx1 sequenced peptides.

**Additional file 3: Figure S2.** Sequence alignment of Tci-Prx1 and closely related nematode typical 2-cys peroxiredoxins.

**Additional file 4: Figure S3.** LPS-responsive reporter ELAM9 cell assay.

## Data Availability

Tci-Prx1 nucleotide coding sequence is available in the NCBI database under the accession number MG972995. The proteomic dataset generated and analysed in the current study is available from the corresponding author upon request.

## Abbreviations

L3: third larval stage
xL3: exsheathed third larval stage
L4: fourth larval stage
Th1: T helper 1
Th2: T helper 2
MD: mucosal-dwelling
LD: lumen-dwelling
ES: excretory–secretory
Prx: peroxiredoxin
2DE: 2D electrophoresis
PDB: Protein Data Bank
CAP: cysteine-rich secretory proteins, antigen 5, and pathogenesis-related 1 proteins
ShK: Stichodactyla toxin
PCR: polymerase chain reaction
IgG: immunoglobulin G
IgA: immunoglobulin A
wt: wild-type
mut: mutant
kDa: kilodalton
IFN-γ: interferon gamma
IL4: interleukin-4
EV: extracellular vesicles
SCP: sperm coating protein-extracellular domain
AAM: alternatively activated macrophages
ROS: reactive oxygen species
PAMP: pathogen-associated molecular pattern molecule
MRC: mannose receptor
HPLC: high performance liquid chromatography
QPCR: quantitative polymerase chain reaction
IPTG: isopropyl-d-1-thiogalactopyranoside
DTT: dithiothretol
