# Supplementary material for: Characterisation of a niche-specific excretory–secretory peroxiredoxin from the parasitic nematode Teladorsagia circumcincta
Source: Parasit Vectors. 2019 Jul 10;12:339. doi: 10.1186/s13071-019-3593-6 (PMC6617597; doi:10.1186/s13071-019-3593-6)

**Additional file 2: Figure S1.** Alignment of Tci-Prx1 sequenced peptides**.** Tci-Prx1 and closely related typical 2-cys peroxiredoxins from *Ovis aries* were aligned using Clustal Omega and MS/MS sequenced Tci-Prx1 peptides were mapped to the alignment (shaded yellow). Aligned regions of host peroxiredoxins are shaded grey. All sequenced peptides distinguish Tci-Prx1 from host *Ovis aries* peroxiredoxins. Sequences included in the alignment are: Tci-Prx1 (MG972995); *Ovis aries* peroxiredoxin 1 (OaPrx1) isoform X1 (XP_011982042); OaPrx1 isoform X2 (XP_004001967); OaPrx2 (NP_001159672); OaPrx4 isoform X1 (XP_004022012); OaPrx4 isoform X2 (XP_011961797).


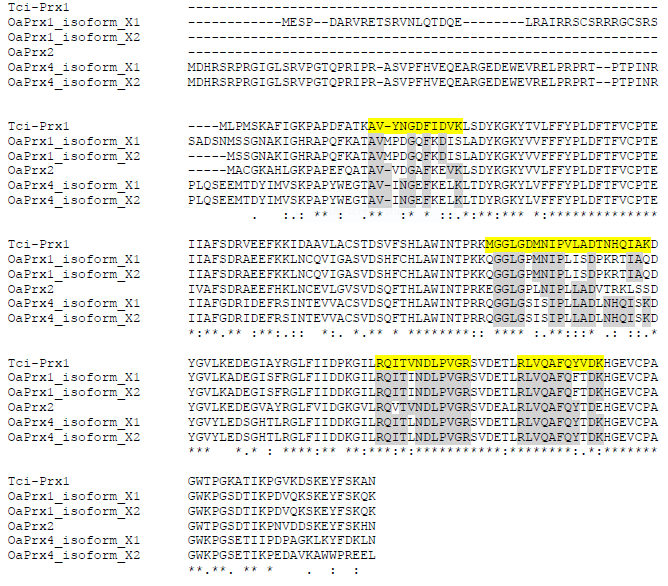

Supplement: Supplementary file 2 — Additional file 2: Figure S1. Alignment of Tci-Prx1 sequenced peptides. [file 13071_2019_3593_MOESM2_ESM.docx]
