# Supplementary material for: Characterisation of a niche-specific excretory–secretory peroxiredoxin from the parasitic nematode Teladorsagia circumcincta
Source: Parasit Vectors. 2019 Jul 10;12:339. doi: 10.1186/s13071-019-3593-6 (PMC6617597; doi:10.1186/s13071-019-3593-6)

**Additional file 3: Figure S2.** Sequence alignment of Tci-Prx1 and closely related nematode typical 2-cys peroxiredoxins. Tci-Prx1 and closely related nematode typical 2-cys peroxiredoxins were aligned using Clustal Omega and shaded to show high sequence conservation (yellow) and low sequence conservation (blue). Amino acid residues conserved across all peroxiredoxin families are boxed in red. Conserved cysteine residues are highlighted (*) and peroxidatic cysteines (C_P_) and resolving cysteines (C_R_) are indicated. Box plot below the alignment shows percentage sequence conservation for each amino acid position. Sequences included in the alignment are: *T. circumcincta* Prx1 (Tci-Prx1, MG972995); *O. ostertagi* Prx (CAD20737); *H. contortus* Prx (CDJ83382); *A. ceylanicum* Prx1 (AcePrx-1, AFP99913); *C. elegans* Prx (NP_001300536).


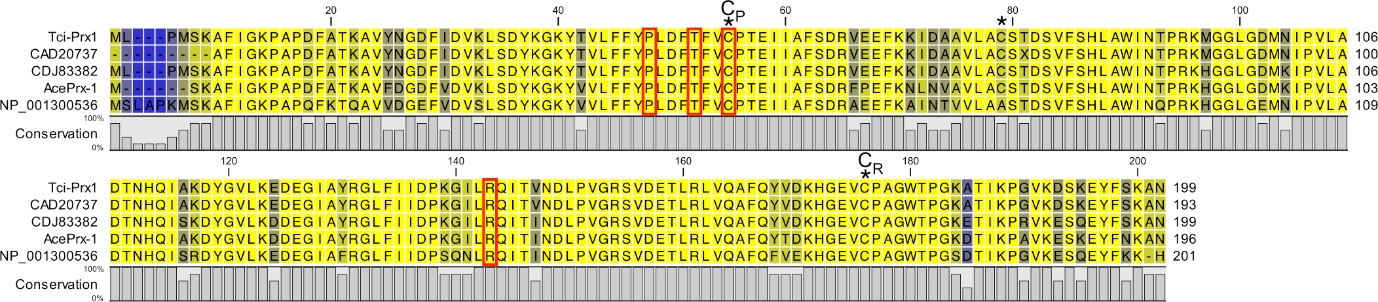

Supplement: Supplementary file 3 — Additional file 3: Figure S2. Sequence alignment of Tci-Prx1 and closely related nematode typical 2-cys peroxiredoxins. [file 13071_2019_3593_MOESM3_ESM.docx]
