# Supplementary material for: Characterisation of a niche-specific excretory–secretory peroxiredoxin from the parasitic nematode Teladorsagia circumcincta
Source: Parasit Vectors. 2019 Jul 10;12:339. doi: 10.1186/s13071-019-3593-6 (PMC6617597; doi:10.1186/s13071-019-3593-6)
Supplement: Supplementary file 4 — Additional file 4: Figure S3. LPS-responsive reporter ELAM9 cell assay. [file 13071_2019_3593_MOESM4_ESM.docx]

**Additional file 4: Figure S3.** LPS-responsive reporter ELAM9 cell assay. Purified recombinant wt Tci-Prx1 and mut Tci-Prx1 did not activate murine macrophage like LPS-responsive reporter ELAM9 cells.

**
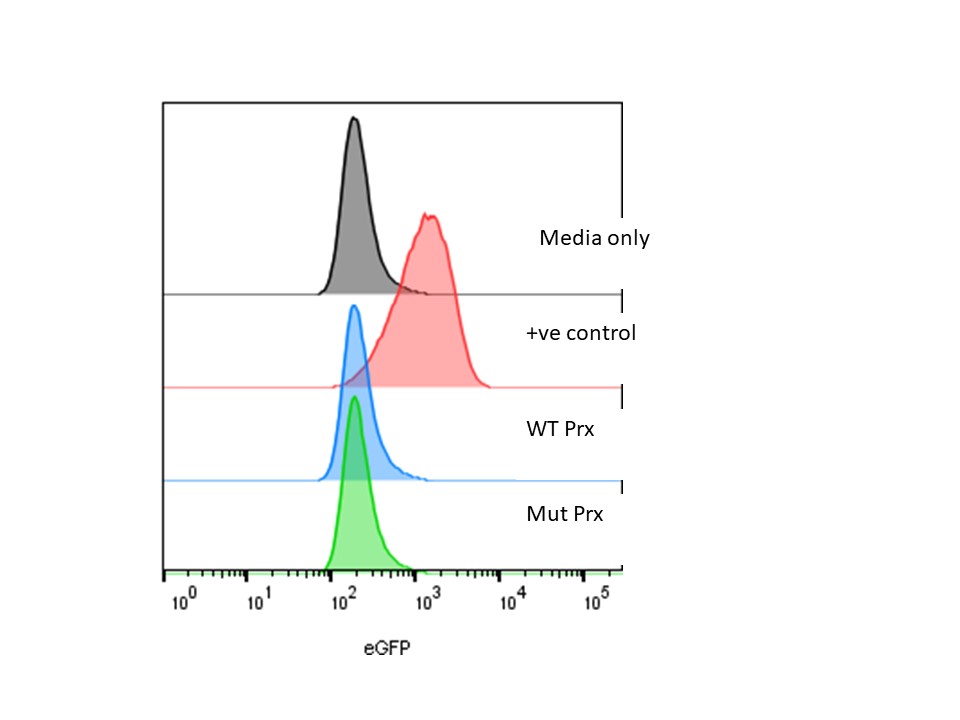
**
